# Comparison of mortality and short-term outcomes between classic, intubation-surfactant-extubation, and less invasive surfactant administration methods of surfactant replacement therapy

**DOI:** 10.3389/fped.2023.1197607

**Published:** 2023-09-15

**Authors:** Seung Yeon Kim, Jiseun Lim, Gyu-Hong Shim

**Affiliations:** ^1^ Department of Pediatrics, Nowon Eulji Medical Center, Eulji University School of Medicine, Seoul, Republic of Korea; ^2^ Department of Preventive Medicine, Eulji University Scholl of Medicine, Daejeon, Republic of Korea; ^3^ Department of Pediatrics, Inje University Sanggye Paik Hospital, Seoul, Republic of Korea

**Keywords:** Intubation-Surfactant-Extubation (InSurE), less invasive surfactant administration (LISA), very-low-birth-weight infants (VLBWIs), surfactant replacement therapy (SRT), mortality, short-term outcomes

## Abstract

**Background:**

Intubation-Surfactant-Extubation (InSurE) and less invasive surfactant administration (LISA) are alternative surfactant replacement therapy methods for reducing the complications associated with invasive mechanical ventilation. This study aimed to compare the Classic, InSurE, and LISA methods in Very-Low-Birth-Weight infants (VLBWIs) in South Korea.

**Methods:**

The Korean Neonatal Network (KNN) enrolled VLBWIs born between January 1, 2019 and December 31, 2020. They were analyzed retrospectively to compare the duration of respiratory support, length of hospitalization, mortality, and short-term outcomes of the three groups.

**Results:**

The duration of invasive ventilator support was shorter in the following order: InSurE (3.99 ± 11.93 days), LISA (8.78 ± 29.32 days), and the Classic group (22.36 ± 29.94 days) (*p* = 0.014, *p* < 0.01) and InSurE had the shortest hospitalization (64.91 ± 24.07 days, *p* < 0.05) although the results couldn't adjust for confounding factor because of irregular distribution. InSurE had the lower risk of intraventricular hemorrhage (IVH) grade II–IV [odds ratio (OR) 0.524 [95% confidence interval (CI): 0.287–0.956], *p* = 0.035] than in the Classic group. Mortality was lower in the InSurE [OR 0.377 (95% CI: 0.146–0.978), *p* = 0.045] and LISA [OR 0.296 (95% CI: 0.102–0.862), *p* = 0.026] groups than in the Classic group. There was a reduced risk of moderate to severe bronchopulmonary dysplasia (BPD) [OR 0.691 (95% CI: 0.479–0.998, *p* = 0.049), OR 0.544 (95% CI: 0.355–0.831, *p* = 0.005), respectively], pulmonary hypertension [OR 0.350 (95% CI: 0.150–0.817, *p* = 0.015), OR 0.276 (95% CI: 0.107–0.713, *p* = 0.008), respectively], periventricular leukomalacia (PVL) [OR 0.382 (95% CI: 0.187–0.780, *p* = 0.008), OR 0.246 (95% CI: 0.096–0.627, *p* = 0.003), respectively], and patent ductus arteriosus (PDA) with treatment [OR 0.628 (95% CI: 0.454–0.868, *p* = 0.005), OR 0.467 (95% CI: 0.313–0.696, *p* < 0.001) respectively] in the InSurE and LISA groups compared to the Classic group.

**Conclusion:**

InSurE showed the lowest duration of invasive ventilator support, length of hospitalization. InSurE and LISA exhibited reduced mortality and decreased risks of moderate to severe BPD, pulmonary hypertension, PVL, and PDA with treatment compared to the Classic group.

## Introduction

Respiratory distress syndrome (RDS) is a progressive disease characterized by a worsening state in the initial hours and days after birth, and is the leading cause of neonatal respiratory morbidity and mortality. In 1980s, the prognosis of RDS improved dramatically with the introduction of a combination of surfactant replacement therapy (SRT) with endotracheal intubation and application of mechanical ventilation (1). Although SRT may reduce the symptom and complications of RDS, the incidence rate of bronchopulmonary dysplasia (BPD) remains high, even though there has been an improvement in survival rates for extremely and very low birth weight preterm infants (2). The Classic SRT involves administering surfactant via an endotracheal tube and applying the mechanical ventilation. This application has been associated with ventilator-induced lung injury (VLI), which may result in BPD, including those infants who have been ventilated very briefly (3). In a pilot study by Victorin et al., instilling exogenous surfactants without mechanical ventilation was considered an alternative to mechanical ventilation (4). Subsequently, Verder et al. introduced the Intubation-Surfactant-Extubation (InSurE) approach, composed of three stages: endotracheal intubation, surfactant administration, and extubation, which can minimize VLI and BPD because it avoids mechanical ventilation (1).

InSurE can help avoid the application of invasive mechanical ventilation in preterm infants who are initially managed with nasal continuous positive airway pressure (nCPAP), which is non-invasive form of ventilation. Studies have shown that InSurE reduces the need for invasive mechanical ventilation and the incidence of BPD (5). However, InSurE has complications, such as injury of the immature lung and pain due to endotracheal intubation, positive pressure ventilation (PPV), and sedation (6). More recently, less invasive surfactant administration (LISA) or minimally invasive surfactant therapy (MIST) has been developed as an alternative for administering surfactant to infants who are spontaneously breathing using a thin catheter inserted into the trachea while applying nCPAP (7, 8). Kribs et al. were the first to evaluate the feasibility of LISA, which administers surfactant into the trachea by direct laryngoscopy via a thin tube with the aid of Magill forceps while the spontaneously breathing infant is supported with nCPAP (9). Following the introduction of this technique, Dargaville et al. described a modification they termed MIST using a semirigid vascular catheter that does not require Magill forceps (10). LISA and MIST have similar methods of surfactant administration, which can avoid the complications of the InSurE and Classic methods; thus, LISA and MIST are currently used interchangeably. LISA is a technique that can avoid the need for endotracheal intubation and PPV and has been shown to reduce the need for mechanical ventilation (7, 11, 12). LISA is expected to reduce lung injury due to barotrauma and volutrauma, thereby preventing the evolution of BPD (12–16).

To summarize, InSurE and LISA, especially LISA, have better outcomes than the Classic SRT. LISA has been performed in Korea since the mid-2010s. The Korean Neonatal Network (KNN), a database of Very-Low-Birth-Weight Infants (VLBWIs) at 22–34 weeks’ gestation admitted in the neonatal intensive care unit (NICU) in South Korea, began to investigate LISA as an SRT in 2019. The current study aims to investigate whether there is a difference in the duration of respiratory support, length of hospitalization, mortality, and short-term outcomes according to the SRT, including the Classic, InSurE, and LISA methods, in VLBWIs in South Korea.

## Materials and methods

### Study design and population

**Figure 1:**
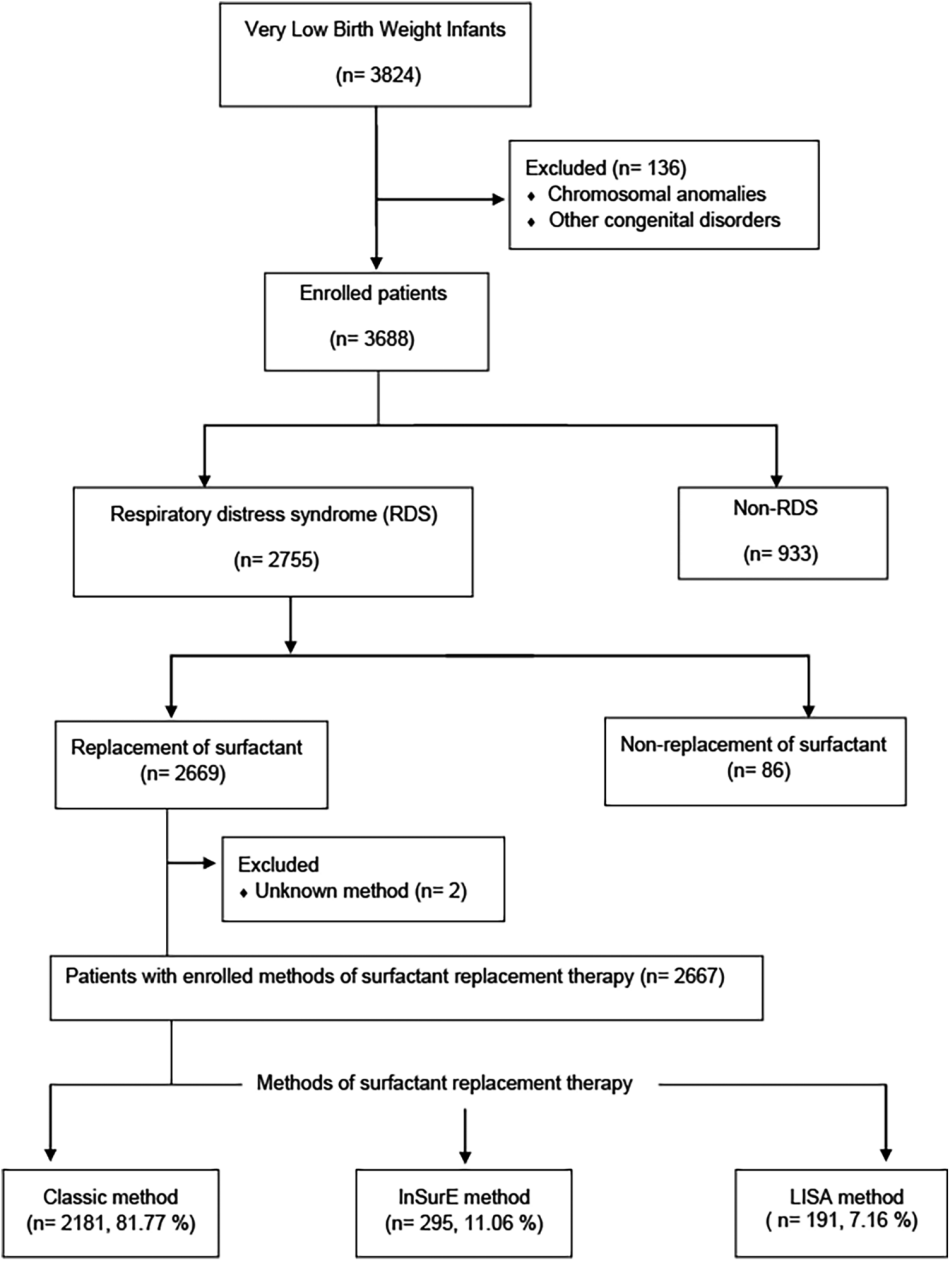
Flowchart of the study population.

The KNN is a prospective cohort registry of VLBWIs admitted to 76 participating NICUs in South Korea. It was launched in 2013 (17) and covers over 80% of VLBWIs in the country. The KNN has established unique systems for data management, including a web-based real-time data display and a site visit monitoring system. Data entry into the network's registry is done by authorized personnel, and institutions must obtain Institutional review boards (IRB) approval and informed consent from parents. The network also employs query generation and external site-visit monitoring for improved data accuracy (17). This study used data that were collected prospectively from the annual reports of 3,824 VLBWIs born between January 1, 2019, and December 31, 2020, as recorded in the KNN registry (Figure 1). Infants with chromosomal anomalies, other congenital disorders, non-RDS, cases of non-replacement of surfactant, and those with unknown SRT methods were excluded. The medical records of VLBWIs who received SRT using the Classic, InSurE, and LISA methods were reviewed for clinical characteristics, perinatal risk factors, duration of respiratory support, length of hospitalization, mortality, and short-term outcomes. Clinical characteristics included gestational age evaluated using the last menstrual period (LMP) or assessed from the time of *in vitro* fertilization, birth weight, sex, 1- and 5-min Apgar scores, need for oxygen at birth, use of PPV at birth, chest compressions at birth, use of nCPAP at birth, number of surfactant administrations, premature rupture of membranes (PROM), outborn status, and postnatal steroid use. Perinatal risk factors included maternal age, diabetes mellitus (DM), hypertension, multiple birth, delivery type, use of antenatal steroids, chorioamnionitis, and use of antibiotics. The respiratory support and hospitalization durations were compared among the three methods. In the InSurE and LISA groups, invasive ventilator support was administered after tracheal intubation by clinician discretion when a fraction of inspired oxygen (FIO_2_) greater than 0.4 was still required after SRT, and when severe or recurrent apnea or persistent respiratory acidosis was observed. The duration of invasive ventilator support was measured in these cases within the InSurE and LISA groups. Additionally, the mortality and short-term outcomes of InSurE and LISA were compared with those of the Classic method, which is the most commonly used method. The following short-term outcomes were investigated: BPD (18), intraventricular hemorrhage (IVH) (19), pulmonary hemorrhage, pulmonary hypertension, air leak syndrome, neonatal necrotizing enterocolitis (NEC) (20), sepsis, periventricular leukomalacia (PVL), retinopathy of prematurity (ROP) (21), and patent ductus arteriosus (PDA) with treatment. Patients who had missing data for specific short-term outcome were excluded from the enrolled patient group to maintain data completeness for each respective outcome. The protocol was approved by the research ethics boards of all clinical centers enrolled in the KNN study, and written informed consent was obtained from each infant's parent or guardian.

### Interventions

The majority of centers enrolled in the current study are implementing SRT in accordance with the standards required for approval by Korean medical insurance, but not protocolized in the KNN cohort data. These standards encompass both therapeutic and prophylactic criteria for SRT approval by Korean medical insurance. The therapeutic SRT criterion stipulates that the patient must exhibit symptoms of respiratory distress (RDS) and characteristic findings of RDS in chest radiographs. Additionally, an FIO_2_ greater than 0.4 is required to maintain the blood oxygen partial pressure within the range of 50–80 mmHg during invasive or noninvasive ventilation. On the other hand, the prophylactic SRT criterion allows for preventive treatment if the birth weight is under 1,250 g or if the gestational age is less than 30 weeks.

The SRT procedure was carried out by skilled neonatal medical professionals specializing in neonatology, with the valuable assistance of neonatal intensive care unit nurses.

#### InSurE

VLBWIs were intubated with a 2.5–3 sized endotracheal tube, and surfactant was administered using a nasogastric catheter (3.5–5 Fr) inserted through the endotracheal tube. After surfactant administration, the endotracheal tube was removed from the infant. If necessary, the infants received nCPAP, nasal intermittent PPV (NIPPV), synchronized NIPPV (sNIPPV), or high-flow nasal cannula (HFNC) after extubation. Note that the KNN cohort data did not include premedication with atropine and sedation with fentanyl before intubation.

#### LISA

A surfactant was instilled using a thin nasogastric catheter (3.5–5 Fr) with the aid of Magill forceps and under direct laryngoscopy. The catheter was inserted into the trachea through the vocal cords, and the surfactant was administered in 3–4 aliquots over 1–3 min, allowing time for airway clearance between each aliquot. Immediately following the surfactant administration, the nasogastric tube was removed. Throughout the procedure, infants with spontaneous breathing continued to receive noninvasive ventilation, such as nCPAP, NIPPV, sNIPPV, or HFNC. The KNN cohort data did not include premedication with atropine during LISA.

### Statistical analysis

Data analysis was performed using SPSS ver. 25.0 (IBM, Armonk, NY, USA) for Windows. We analyzed clinical characteristics and perinatal risk factors with a Pearson's chi-square test or one-way ANOVA. Values with *p < *0.05 were considered statistically significant. Kruskal–Wallis tests were used to compare the duration of respiratory support and length of hospitalization in the NICU, and *post hoc* analysis was performed via pairwise comparison. A multivariate binary logistic regression model was used to compare the short-term outcomes and mortality of the Classic, InSurE, and LISA methods. The Classic method was designated the reference method. Confounding factors included clinical characteristics and perinatal risk factors that significantly differed among the three SRT groups. Adjusted survival curves for each SRT type were drawn using Cox proportional hazard analysis with the PROC PHREG process (22) in SAS Ver. 9.4 (SAS Institute Inc., Cary, NC, USA).

## Results

Of the 3,824 VLBWIs born during the study period, 1,157 were excluded for chromosomal anomalies, other congenital disorders, non-RDS, non-replacement of surfactant, or unknown SRT methods. The remaining 2,667 infants were enrolled, including 2,181 (81.77%) with the Classic method, 295 (11.06%) with InSurE, and 191 (7.16%) with LISA (Figure 1).

### Clinical characteristics and perinatal risk factors

The clinical characteristics and perinatal risk factors of the study population are as shown in Table 1. The mean gestational age (weeks) of the Classic, InSurE, and LISA groups was 27.41 ± 2.49 weeks, 29.35 ± 1.57 weeks, and 28.78 ± 1.75 weeks, respectively, and these differences were statistically significant. The mean birth weight (grams) of the Classic, InSurE, and LISA groups was 962.53 ± 280.79, 1,180.96 ± 209.18, and 1,106.31 ± 226.93, respectively, and these differences were significant. There was a significant difference in sex ratios between the groups, with a lower proportion of male infants in the InSurE group than in the Classic and LISA groups. Maternal factors such as age, DM, hypertension, and multiple births significantly differed between the groups. The clinical characteristics of VLBWIs, including 1- and 5-minute Apgar scores, PPV at birth, chest compression at birth, nasal CPAP at birth, number of SRT administrations, PROM, antenatal steroid use, chorioamnionitis, antenatal antibiotics use, and postnatal steroid use, also differed significantly between the groups. Specifically, most of the clinical data at birth were worse in the Classic group than in the InSurE and LISA groups.

**Table 1:** Clinical characteristics and perinatal risk factors.

Variables	Methods of surfactant administration (*n* = 2,667)			*p-*value
	Classical method (*n* = 2,181)	InSurE method (*n* = 295)	LISA method (*n* = 191)	
Gestational weeks, mean ± SD	27.41 ± 2.49	29.35 ± 1.57	28.78 ± 1.75	<0.001*
Birth weight, g, mean ± SD	962.53 ± 280.79	1,180.96 ± 209.18	1,106.31 ± 226.93	<0.001*
Males, *n* (%)	1,121 (51.4)	128 (43.2)	98 (51.3)	0.035*
Maternal age, mean ± SD	33.76 ± 4.45	34.39 ± 4.43	34.44 ± 4.10	0.014*
Maternal DM, *n* (%)	270 (12.4)	58 (19.7)	20 (10.5)	0.001*
Maternal hypertension, *n* (%)	445 (20.4)	80 (27.1)	57 (29.8)	0.001*
Multiple birth, *n* (%)	832 (38.1)	132 (44.7)	90 (47.1)	0.008*
Delivery type, cesarean, *n* (%)	1,771 (81.2)	253 (85.4)	163 (85.3)	0.094
1-minute Apgar score (min-max)	4 (0–9)	6 (1–9)	6 (1–10)	<0.001*
5-minute Apgar score (min-max)	6 (0–10)	8 (2–10)	8 (4–10)	<0.001*
Need for oxygen at birth	2,077 (96.6)	240 (94.5)	166 (94.3)	0.093
Positive pressure ventilation at birth, *n* (%)	2,006 (93.3)	186 (73.2)	119 (67.6)	<0.001*
Chest compression at birth, *n* (%)	107 (5.0)	1 (0.4)	0 (0)	<0.001*
Nasal CPAP at birth, *n* (%)	491 (22.8)	112 (44.1)	103 (58.5)	<0.001*
Number of SRT administrations, mean ± SD	1.44 ± 0.71	1.16 ± 0.42	1.37 ± 0.64	<0.001*
Premature rupture of membrane, *n* (%)	881 (40.4)	96 (32.5)	48 (25.1)	<0.001*
Outborn, *n* (%)	51 (2.3)	7 (2.4)	2 (1.0)	0.508
Use of antenatal steroid, *n* (%)	1,899 (87.1)	276 (93.6)	181 (94.8)	<0.001*
Chorioamnionitis, *n* (%)	732 (33.6)	53 (18.0)	40 (20.9)	<0.001*
Use of antenatal antibiotics, *n* (%)	1,441 (66.1)	195 (66.1)	87 (45.5)	<0.001*
Postnatal steroid use, *n* (%)	795 (36.5)	26 (8.8)	37 (19.4)	<0.001*

SD, standard deviation; InSurE, Intubation-Surfactant-Extubation; LISA, less invasive surfactant administration; DM, diabetes mellitus; CPAP, continuous positive airway pressure; SRT, surfactant replacement therapy.

* *p-*value < 0.05.

### Duration of respiratory support and length of hospitalization

The duration of respiratory support and length of hospitalization are as shown in Table 2. The respiratory support duration, including invasive ventilator support and oxygen supply, significantly differed between the groups. The duration of invasive ventilator support (days) was shortest in the InSurE group (3.99 ± 11.93) compared with the Classic (22.36 ± 29.94, *p* < 0.001) and LISA (8.78 ± 29.32, *p* = 0.014) groups. Moreover, the duration of invasive ventilator support was shorter in the LISA group than in the Classic group (*p* < 0.001). There was no significant difference between the three groups in the duration of noninvasive ventilator support (*p* = 0.271). The oxygen supply duration (days) in the Classic, InSurE, and LISA groups was 6.74 ± 11.97, 2.97 ± 8.83, and 5.98 ± 11.70, respectively. The oxygen supply duration was the shortest in the InSurE group, but there was only a significant difference between the InSurE and Classic methods *(p* < 0.001). There was no significant difference between the three groups in the duration of HFNC (*p* = 0.768).

**Table 2:** Duration of respiratory support and length of hospitalization in NICU.

Variables (days, mean ± SD)	Classical method (*n* = 2,181)	InSurE method (*n* = 295)	LISA method (*n* = 191)	*p*-value	Pairwise comparisons		
					InSurE-LISA	InSurE-Classical	LISA-Classical
Duration of invasive ventilator support	22.36 ± 29.94	3.99 ± 11.93	8.78 ± 29.32	<0.001*	0.014*	<0.001*	<0.001*
Duration of noninvasive ventilator support	26.44 ± 24.41	24.78 ± 19.72	26.95 ± 20.63	0.271			
Duration of oxygen supply	6.74 ± 11.97	2.97 ± 8.83	5.98 ± 11.70	<0.001*	0.083	<0.001*	0.104
Duration of high flow nasal cannula	15.31 ± 16.84	13.67 ± 14.03	13.20 ± 13.66	0.768			
Length of hospitalization	79.68 ± 48.87	64.91 ± 24.07	75.91 ± 36.80	<0.001*	0.005*	<0.001*	0.379

SD, standard deviation; InSurE, Intubation-Surfactant-Extubation; LISA, less invasive surfactant administration.

* *p-*value < 0.05.

The length of hospitalization (days) in the Classic, InSurE, and LISA groups was 79.68 ± 48.87, 64.91 ± 24.07, and 75.91 ± 36.80, respectively. There was a significant difference between the InSurE and LISA groups and between the InSurE and Classic groups. The InSurE group had the shortest length of hospitalization.

### Mortality and short-term outcomes

**Figure 2:**
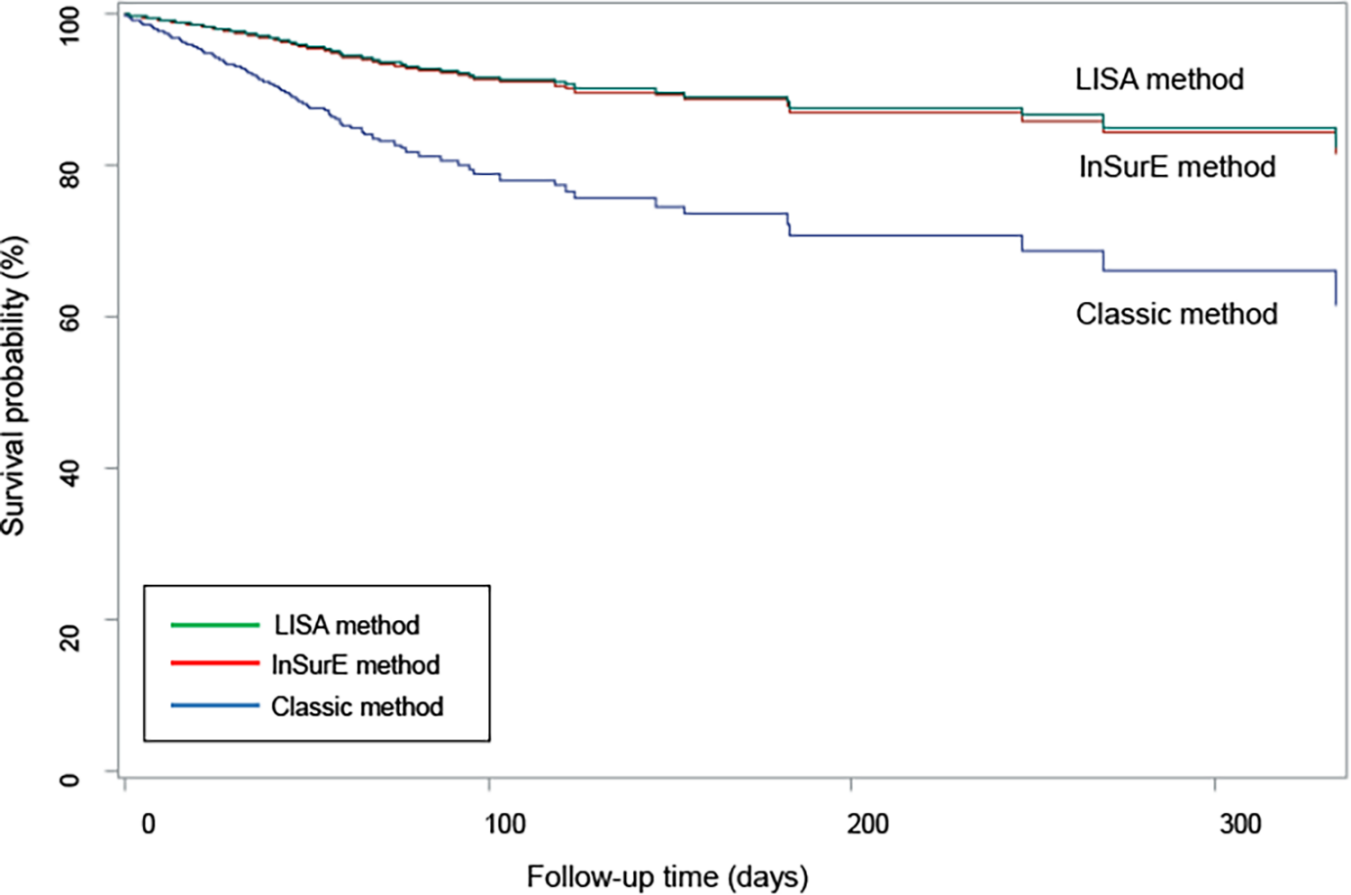
Adjusted survival curve for classic, inSurE, and LISA method group.

The mortality and short-term outcomes of the Classic group were compared with those of the InSurE and LISA groups; the Classic group was the reference. Table 3 shows mortality and short-term outcomes comparing the Classic and InSurE groups, as well as comparing the Classic and LISA groups. Mortality adjusted for confounding factors such as gestational week, birth weight, sex, maternal age, maternal DM, maternal hypertension, multiple birth, 1 and 5-min Apgar scores, PPV at birth, chest compression at birth, nCPAP at birth, number of SRT administrations, PROM, use of antenatal steroid, chorioamnionitis, use of antenatal antibiotics, and postnatal steroid use was lower with InSurE {odds ratio (OR) 0.377 [95% confidence interval (CI): 0.146–0.978], *p* = 0.045} and LISA [OR 0.296 (95% CI: 0.102–0.862), *p* = 0.026] than with the Classic method. The survival curve for the three groups after adjusting for the same confounding factors is shown in Figure 2. The LISA and InSurE groups showed superior survival rates than the Classic group during all observed periods. The risk of moderate to severe BPD was lower in the InSurE [OR 0.691 (95% CI: 0.479–0.998), *p* = 0.049] and LISA [OR 0.544 (95% CI: 0.355–0.831), *p* = 0.005] groups than in the Classic group. The risk of IVH grade II–IV was lower in the InSurE group than in the Classic group [OR 0.524 (95% CI: 0.287–0.956), *p* = 0.035]. However, there was no significant difference in the risk of IVH grade II–IV between the LISA and Classic groups [OR 0.837 (95% CI: 0.473–1.481), *p* = 0.541]. The risk of pulmonary hypertension was lower with InSurE [OR 0.35 (95% CI: 0.150–0.817), *p* = 0.015] and LISA [OR 0.276 (95% CI: 0.107–0.713), *p* = 0.008] than with the Classic method. The PVL risk was lower in the InSurE [OR 0.382 (95% CI: 0.187–0.780), *p* = 0.008] and LISA [OR 0.246 (95% CI: 0.096–0.627), *p* = 0.003] groups than in the Classic group. The risk of PDA with treatment was lower in the InSurE [OR 0.628 (95% CI: 0.454–0.868), *p* = 0.005] and LISA [OR 0.467 (95% CI: 0.313–0.696), *p* < 0.001] groups than in the Classic group. There were no significant differences in risk of pulmonary hemorrhage, air leak syndrome, NEC grade II or higher, sepsis, and ROP with treatment between the InSurE and Classic groups and between the LISA and Classic groups.

**Table 3:** Mortality and short-term outcomes comparison between classic and inSurE method or classic and LISA method.

Variables	Classic method (*n* = 2,181)		InSurE method (*n* = 295)			LISA method (*n* = 191)	
	Applicable/enrolled patient *n* (%)	Applicable/enrolled patient *n* (%)	AOR^a^ (95% CI)	*p-*value	Applicable/enrolled patient *n* (%)	AOR^a^ (95% CI)	*p-*value
Mortality	390/2,181 (17.9)	6/295 (2.0)	0.377 (0.146–0.978)	0.045*	4/191 (2.1)	0.296 (0.102–0.862)	0.026*
BPD, ≥ moderate	874/1,761 (49.6)	61/291 (21.0)	0.691 (0.479–0.998)	0.049*	51/187 (27.3)	0.544 (0.355–0.831)	0.005*
IVH grade ≥ grade Ⅱ	510/2,085 (24.5)	14/294 (4.8)	0.524 (0.287–0.956)	0.035*	16/191 (8.4)	0.837 (0.473–1.481)	0.541
PH	160/2,021 (7.9)	9/295 (3.1)	0.937 (0.428–2.051)	0.870	5/191 (2.6)	0.473 (0.164–1.365)	0.166
PHN	376/2,181 (17.2)	7/295 (2.4)	0.350 (0.150–0.817)	0.015*	7/191 (3.7)	0.276 (0.107–0.713)	0.008*
Air leak syndrome	130/2,181 (6.0)	5/295 (1.7)	0.679 (0.262–1.764)	0.427	6/191 (3.1)	1.062 (0.431–2.618)	0.895
NEC ≥ grade Ⅱ	174/2,181 (8.0)	12/295 (4.1)	1.015 (0.526–1.959)	0.964	14/191 (7.3)	1.234 (0.641–2.373)	0.529
Sepsis	477/2,181 (21.9)	45/295 (15.3)	1.328 (0.900–1.959)	0.153	31/191 (16.2)	0.858 (0.536–1.373)	0.523
PVL	220/2,084 (10.6)	9/294 (3.1)	0.382 (0.187–0.780)	0.008*	5/191 (2.6)	0.246 (0.096–0.627)	0.003*
ROP with treatment	267/2,181 (12.2)	6/295 (2.0)	0.544 (0.229–1.295)	0.169	12/191 (6.3)	0.937 (0.454–1.932)	0.859
PDA with treatment	887/2,181 (40.7)	78/295 (26.4)	0.628 (0.454–0.868)	0.005*	41/191 (21.5)	0.467 (0.313–0.696)	<0.001*

A multivariate binary logistic regression model was used and the Classic method was the reference group.

AOR, adjusted odds ratio; CI, confidence interval; InSurE, Intubation-Surfactant-Extubation; LISA, less invasive surfactant administration; BPD, Bronchopulmonary dysplasia; IVH, Intraventricular hemorrhage; PH, pulmonary hemorrhage; PHN, pulmonary hypertension; NEC, necrotizing enterocolitis; PVL, periventricular leucomalacia; ROP, retinopathy of prematurity; PDA, patent ductus arteriosus.

^a^ Odds ratios and *p*-values adjusted for clinical characteristics and perinatal risk factors that significantly differed among the three surfactant replacement therapy groups.

* *p*-value < 0.05.

## Discussion

In the current study, the duration of invasive ventilator support and length of hospitalization were higher in the Classic group than in the InSurE and LISA groups as expected. However, InSurE had a shorter duration of invasive ventilator support and length of hospitalization of NICU than LISA, although LISA was introduced as an alternative to compensate for the complication of InSurE. In addition, the risk of IVH grade II–IV was lower in the InSurE group than in the Classic group while there was no significant difference in the risk of IVH grade II–IV between the LISA and Classic groups. LISA has shown better results than InSurE in reducing the incidence of BPD and the need for mechanical ventilation (12, 23–25). The current study revealed distinct outcomes. The InSurE group demonstrated more favorable results in terms of both the duration of invasive ventilator support and the length of hospitalization compared to the LISA group. In our country, the InSurE method is more commonly practiced, while the LISA method is still being introduce. We suspect that this difference in familiarity may have influenced the observed results. However, numerous studies have compared LISA and InSurE for preterm infants, and the findings suggest that there are no significant differences between these two approaches (26–29). Gupta et al. found no disparities in the need for invasive mechanical ventilation in the first 72 h of life, hemodynamically significant PDA, IVH (>grade 2), BPD, and combined outcome of BPD/mortality when comparing MIST, which is used interchangeably with LISA, and InSurE (26). Similarly, Kaniewska et al. reported that there were no significant differences between LISA and InSurE groups in terms of the need for intubation, duration of mechanical ventilation, duration of oxygen supplementation, and the incidence of BPD (27). Pareek et al. also observed no statistically significant differences in major complication rates, duration of respiratory support, hospital stay, and mortality between LISA and InSurE groups (28). Likewise, Xu et al. found no significant differences in the incidence of BPD, severity of BPD at 36 weeks postmenstrual age, and the rate of mechanical ventilation within 72 h after birth when comparing the LISA and InSurE groups (29). Therefore, the superiority of LISA over InSurE remains controversial. There is emerging caution regarding LISA due to the lack of a clear pathophysiological background and uncertain clinical benefits (30, 31). In addition, LISA has the following disadvantages: differences in sedation policies, unstandardized surfactant dose, unclear pressure for spreading surfactant, unclear oxygen concentration during the procedure, the potential need for multiple attempts at catheter placement, and 5%–40% incidence of gagging, bradycardia, apnea, desaturations, and decreased regional cerebral oxygenation (30, 32, 33). Recently, Dargaville et al. reported in a large-scale randomized controlled trial that MIST, which is used interchangeably with LISA, did not significantly reduce the incidence of the composite outcome of death or BPD at 36 weeks’ postmenstrual age in preterm infants with a gestational age of 25–28 weeks (34). Additional large-scale randomized controlled trials are needed to address the limitations of LISA.

After adjusting for confounding factors, including clinical characteristics and perinatal risks that differed significantly among the three groups, both InSurE and LISA demonstrated better outcomes than the Classic method. These outcomes included lower risks of death and moderate to severe BPD, which are important outcomes of SRT, and lower risks of pulmonary hypertension, PVL, and PDA with treatment. Our data show that the Classic method remains the dominant SRT in South Korea, although it has the worst clinical characteristics and perinatal risk factors. It is possible that because it is the more familiar method, it is considered to be more stable and safer. However, the current study found that, after adjusting for confounding factors, mortality and the risk of short-term outcomes were lower in the LISA and InSurE groups than in the Classic group. New modes of surfactant delivery, such as InSurE and LISA, can decrease mechanical ventilation and BPD rates (3, 5, 35, 36). In an appropriate group of spontaneously breathing infants, transitioning from the Classic method to InSurE or LISA for SRT might be necessary in South Korea.

This study has some limitations. First, it was a retrospective study despite using nationwide data. Second, due to the non-normal distribution of results, it was not possible to perform a linear regression analysis to adjust for perinatal risk factors and clinical data with statistically significant differences in order to compare the duration of respiratory support and length of hospitalization between the three SRT groups. Moreover, the infants who received the Classic method exhibited higher baseline severity compared to the group that underwent SRT using the InSurE and LISA methods. Consequently, the possibility that the results of Table 2 are due to these basic characteristics cannot be disregarded, necessitation an interpretation of the results with this consideration in mind. Lastly, there was no standardized rule for LISA, such as a policy of sedation, need for oxygen, type of noninvasive ventilator, and surfactant dose, among the enrolled KNN data.

Despite these limitations, to the best of our knowledge, this is the first study to compare the duration of respiratory support, length of hospitalization, mortality, and short-term outcomes among SRT methods, including the Classic, InSurE, and LISA methods, with nationwide data in South Korea. In addition, it is meaningful that the results were derived by correcting all confounding variables, such as clinical characteristics and perinatal risk factors, which showed statistically significant differences in mortality and short-term outcomes of three groups with differences in clinical characteristics and perinatal risk factors.

## Conclusions

The duration of respiratory support significantly differed among the Classic, InSurE, and LISA methods, with InSurE demonstrating the shortest duration for invasive ventilator support. InSurE also resulted in significantly shorter hospitalization compared to the Classic method and LISA. However, it's important to note that the aforementioned results could be influenced by the statistical limitations arising from the non-normal distribution of the data. Adjusting for confounding factors, such as clinical characteristics and perinatal risk factors that varied significantly among the three groups, revealed that InSurE and LISA were associated with significantly lower risks of death, moderate to severe BPD, pulmonary hypertension, PVL, and PDA with treatment compared to the Classic method.

## Data Availability

The original contributions presented in the study are included in the article/Supplementary Material, further inquiries can be directed to the corresponding authors.
